# Biomimetic Approach to Counter Streptococcus mutans Biofilm: An In Vitro Study on Seashells

**DOI:** 10.7759/cureus.47758

**Published:** 2023-10-26

**Authors:** Annie Sylvea Valan, Jogikalmat Krithikadatta, Mukesh Doble, M Lakshmipathy

**Affiliations:** 1 Conservative Dentistry and Endodontics, Saveetha Dental College and Hospital, Saveetha Institute of Medical and Technical Sciences, Saveetha University, Chennai, IND; 2 Cariology, Saveetha Dental College and Hospital, Saveetha Institute of Medical and Technical Sciences, Saveetha University, Chennai, IND

**Keywords:** surface morphology, seashells, periostracum, fourier transform infrared spectroscopy, atomic force microscopy, antibiofilm assay

## Abstract

Aim

This study aimed to investigate the anti-adherent property of the seashell surface and periostracum to prevent the formation of *Streptococcus mutans* biofilm*.*

Materials and methods

The seashells were initially collected from the natural urban beach, and an antibiofilm assay of the shells with and without periostracum was performed against *Streptococcus mutans*. Furthermore, the seashells were analyzed with a stylus profilometer (Mitutoyo Surftest SJ-301, Mitutoyo America Corporation, Illinois, USA), atomic force microscope (AFM; Nanosurf Easyscan 2, Nanosurf Inc., USA), contact angle assessment, Fourier-transform infrared (FTIR) spectroscopy analysis, and scanning electron microscopy (SEM; JEOL USA, Inc., FE-SEM IT800, Massachusetts, USA) analysis. The ability of seashells to prevent the attachment of *Streptococcus mutans* and form a biofilm with and without periostracum was studied by crystal violet assay.

Results

The results revealed that shells without periostracum promoted higher biofilm formation when compared to those having intact periostracum (by 15%, p<0.001). Shell 1 showed the highest biofilm formation, whereas shell 3 showed the least biofilm formation due to the differences in their surface morphologies. The remaining shells (4, 2, 6, and 5) showed interspersed biofilm formation.

Conclusion

In summary, our study was able to correlate the topologies of the shell surface with the biofilm formed by *Streptococcus mutans* with the wetting behavior of those shell surfaces and their roughness. More hydrophobic surfaces (with intact periostracum) were observed to lead to less attachment (correlation coefficient=-0.67). This study can pave the way for designing such biomimetic surfaces to prevent bacterial attachment.

## Introduction

A biofilm constitutes a meticulously organized arrangement comprising bacterial cells enveloped within an extracellular polymeric matrix, autonomously generated by the bacteria, and firmly attached to a surface. Apart from extracellular polysaccharides, extracellular proteins and extracellular DNA are also present in biofilms. These biofilms can also be conceptualized as a condensed layer of microbiota or as a community derived from microorganisms, consisting of cells permanently affixed to a surface, interface, or each other [1]. The formation of biofilms remains one of the main strategies for any bacterial community to escape from the biological effect of any antimicrobial agent. Indeed, these antimicrobial drugs are absorbed more slowly by bacterial cells present in biofilms than they are in their planktonic condition because of the tight entanglement of the polymeric matrix. Furthermore, the biofilm creates an optimal environment for microbial cells to initiate genetic mutations, thereby increasing their likelihood of survival, long-term presence, and developing resistance to antimicrobial agents [2]. In dentistry, encountering biofilms poses a great challenge, either due to the recurrence of the infection or treatment failure. More specifically, the root canal treatment procedure necessitates access opening of the root canal and disinfecting the canal space using antiseptics. This mechanism guarantees the elimination of vital or decayed tissue remnants, the eradication of the organisms within the root canal system, the removal of the microbial biofilm, and the elimination of the hard tissue debris generated during root canal instrumentation [3]. The ideal objective of endodontic research has been to develop techniques that can totally eradicate the bacterial biofilm.

There is an increasing need for more cytocompatible and dentin-friendly compounds to overcome the drawbacks of current chemical antibacterial agents. Due primarily to their biocompatibility, herbal products are becoming increasingly popular across all medical specialties. The medical benefits of herbal extracts, including their anti-inflammatory, antimicrobial, and antioxidant activities, favor using them in endodontics for disinfecting canals. Multiple research investigations have been conducted to assess various natural products for their potential use as irrigants and/or intra-canal medications [4].

In recent years, biomimetic properties and their application in biomedical and clinical fields have largely been explored. The phenomenon known as "fouling" (or "biofouling") pertains to the colonization of a substrate by an opportunistic organism (or multiple species of colonizing organisms) and has undergone thorough investigation and characterization by various researchers [5]. Biofouling of fishing nets and ship hulls previously dealt with antifouling agents were made to resist fouling using periostracum (a tough but pliable, proteinaceous covering of the mussel shell). Studies have shown that removing periostracum from the shell of *Mytilus edulis* mussels resulted in an elevated settlement of barnacles and algae on the shell surface [6]. However, mussels possessing an intact periostracum demonstrated increased resistance to fouling pressure [7].

Conversely, reports have highlighted the advantages of microtopography on mussel shells as an effective physical deterrent against fouling [8,9]. Marine organisms, including sharks and mollusks, possess micro-textured surfaces. Despite coexisting in the same environment as human-made structures quickly colonized by biofouling organisms, these natural surfaces remain fouling-free [8]. Certainly, the exact mechanisms underlying the resistance properties conferred by periostracum, which prevent the settlement of biofilm-forming organisms, remain ambiguous and require further elucidation. The potential application of periostracum in dentistry, specifically its resistance properties, has not been evaluated. Therefore, this study aims to assess the anti-adherent properties of periostracum against potent biofilm-forming oral pathogens.

## Materials and methods

This research was conducted after getting approval from the Institutional Ethics Committee (IHEC/SDC/ENDO-2103/22/178).

Sample preparation

Seashells were initially collected from the natural urban beach in Chennai, Tamil Nadu, India, based on colors, shapes, and decorative patterns for our study and collection of periostracum. Six pairs of seashells of different surface texture were selected. The shells were washed with running tap water to remove any debris or dirt and then washed gently using sterile distilled water. The shells were segregated into two categories: the test and control groups. The test group consisted of shell samples with periostracum removed, while the control group contained samples with an intact periostracum.

Removal of periostracum

Periostracum (a protein layer present on the shell) of the collected shells was removed by adopting the method of Grandison et al. [10]. To ease the removal of periostracum, the seashells were immersed in a vinegar and seawater solution (1:2), taken in a 250 mL beaker, and left undisturbed for 24 hours. Periostracum was removed from the shell with the help of sterile forceps and retained for further experimental [11].

Sectioning of shells

Both the test and control groups of shells were sectioned to a dimension of 5 x 5 mm with the help of a diamond disc. They were autoclaved prior to the initiation of the test.

Preparation of inoculum

One hundred milliliters of brain heart infusion broth prepared in a 250 mL Erlenmeyer flask was inoculated with four to five individual colonies of *Streptococcus mutans* (sourced from the Department of Microbiology, Dr. ALM PG Institute of Basic Medical Sciences, Chennai) from the Petri plate and incubated overnight at 37°C. The growth of the bacterium was evidenced by the broth's turbidity and used for biofilm assay.

Antibiofilm assay

The sectioned shells were carefully placed in a 96-well plate using sterile forceps, and then each sample was introduced to 150 μL of broth culture. Subsequently, the samples were incubated for 48 hours at 37℃. After incubation, the broth was aspirated from the wells using a sterile pipette and washed with phosphate-buffered saline (PBS) solution. Then 150 μL of crystal violet (0.2%) was added to each well containing shells and allowed to stand for 15-20 minutes. This was followed by removing the dye and washing with PBS to remove unbound and excess dye. Then the dye was dissolved by adding 150 μL of glacial acetic acid (30%) in each well. Readings were taken using an ELISA plate reader at 570 nm, and the absorbance value was recorded.

Scanning electron microscopy analysis

The shell samples were mounted in stubs using carbon tape for stabilization. They were then sputter-coated using platinum sputter coating and observed with a field emission scanning electron microscopy (SEM; JEOL USA, Inc., FE-SEM IT800, Massachusetts, USA) at 3.00 kV.

Fourier-transform infrared spectroscopy analysis

A Fourier-transform infrared (FTIR) spectroscopy analysis was done using powdered periostracum samples with the help of an infrared spectroscope.

Atomic force microscopic analysis

An atomic force microscopic (AFM; Nanosurf Easyscan 2, Nanosurf Inc., USA) analysis was used to observe three-dimensional and topographical details of the shell samples to ascertain the roughness of the surface. The assessment of the surface roughness of the shells involved the calculation of key parameters, including the average roughness (Sa), root mean square roughness (Sq), and the mean difference between the highest peaks and lowest valleys (Sz) using the Nanosurf C3000 software [12].

Surface roughness profiling

A stylus profilometer (Mitutoyo Surftest SJ-301, Mitutoyo America Corporation, Illinois, USA) was used to assess the surface roughness of the shell samples. On the sample surface, random points were marked as measurement locations for roughness [13]. The surface profile was determined and expressed in terms of mean roughness (Ra), root mean square (Rq), and the mean difference between the highest peaks and lowest valleys (Rz). The roughness profile of the samples was performed in triplicates.

Surface tension measurement

A sessile drop method using an Ossila goniometer (Ossila Ltd, Sheffield, UK) was used to assess the contact angle of the selected shells. The system was calibrated by focusing on a sphere with a known width. Once the calibration was done, the sample was placed in the center of the vertical tilt stage, and the stage height was adjusted until the sample was seen in the bottom half of the image. The droplet was dispensed using a micro syringe, and the software detected the edges of the droplet within the ROI and showed the mean average of left and right angles.

Statistical analysis

A two-way ANOVA was performed using SPSS Statistics version 23.0 (IBM Corp. Released 2015. IBM SPSS Statistics for Windows, Version 23.0. Armonk, NY: IBM Corp.) to determine the statistical differences in the biofilm formation between various shells and with and without periostracum.

## Results

**Figure 1:**
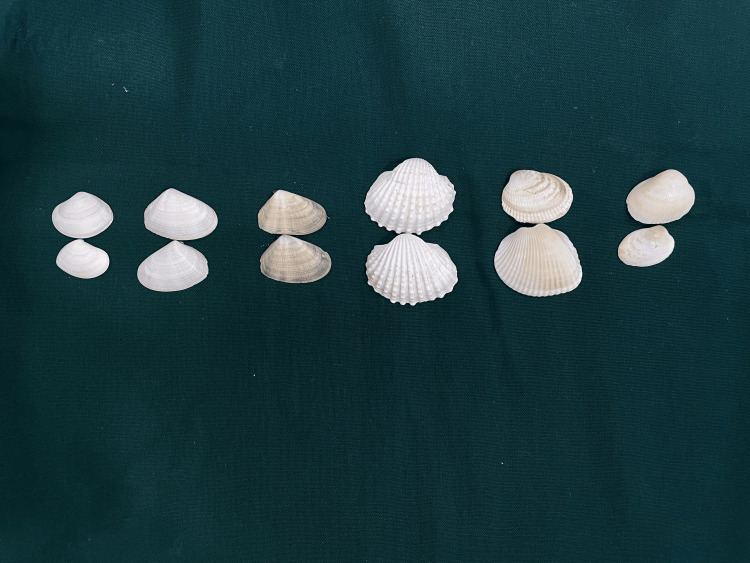
Seashells (S1 to S6 from left to right, respectively) with different color, texture, and ridges

Seashells of different surface textures collected from the natural urban beach in Chennai are shown in Figure 1. Some shells have fine ridges, and a few have coarse surfaces.

Antibiofilm assay

**Figure 2:**
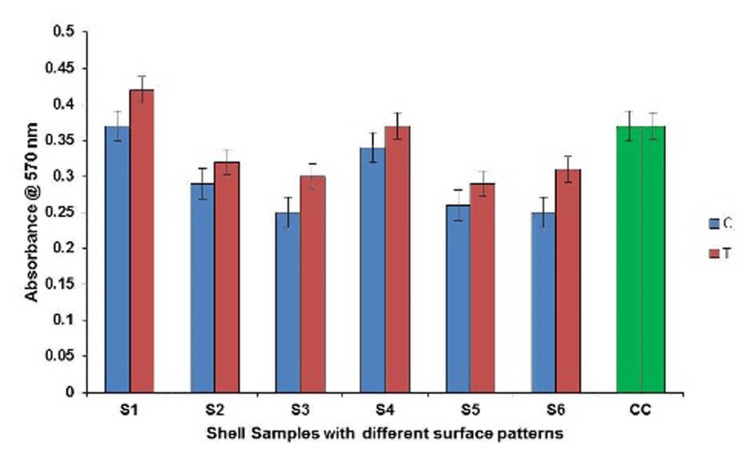
The anti-adherent property of periostracum intact (C) and periostracum removed (T) shells with different surface patterns incubated with Streptococcus mutans. Control group (blue) - intact periostracum. Test group (red) - without periostracum. CC - cell control without any treatment

This study investigates the antibiofilm efficacy of periostracum extracted from the seashells collected from the urban shoreline of Marina Beach located inside the city. When observed by crystal violet assay, the antibiofilm property of the shells with and without periostracum revealed that shells without periostracum promoted higher biofilm formation on par with those with intact periostracum (Figure 2). Shell 1 showed the highest biofilm formation, whereas shell 3 showed the least. The remaining shells (4, 2, 6, and 5) showed interspersed biofilm formation, which may be due to the surface architecture of the shells. The influence of the patterns of shells 1 and 3, representative of the smooth and rugged patterns, in improving the adhesion or inhibition of biofilm was retained for the experiment. These shells were subjected to roughness profiling, SEM, and AFM analyses.

SEM analysis

**Figure 3:**
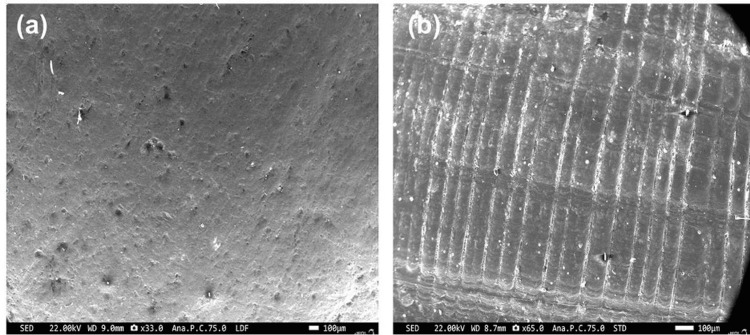
SEM micrograph of shells (a) 1 and (b) 3, exhibiting different surface morphologies at a scale bar of 100 microns

The surface morphology of shells 1 and 3 was analyzed using electron microscopy. The electron micrograph of shell 1 (Figure 3a) showed a smooth surface interspersed with submicron- to nanometer-sized pores contributing a large surface area. On the other hand, shell 3 (Figure 3b) demonstrated a consistent pattern of ripple-like microstructures extending uniformly parallel across the entire shell, devoid of any branching. This difference in the microarchitecture of shells 1 and 3, representative of the smooth and rugged patterns, served as classic examples of surface modification for promoting or impeding biofilm formation.

AFM analysis

**Figure 4:**
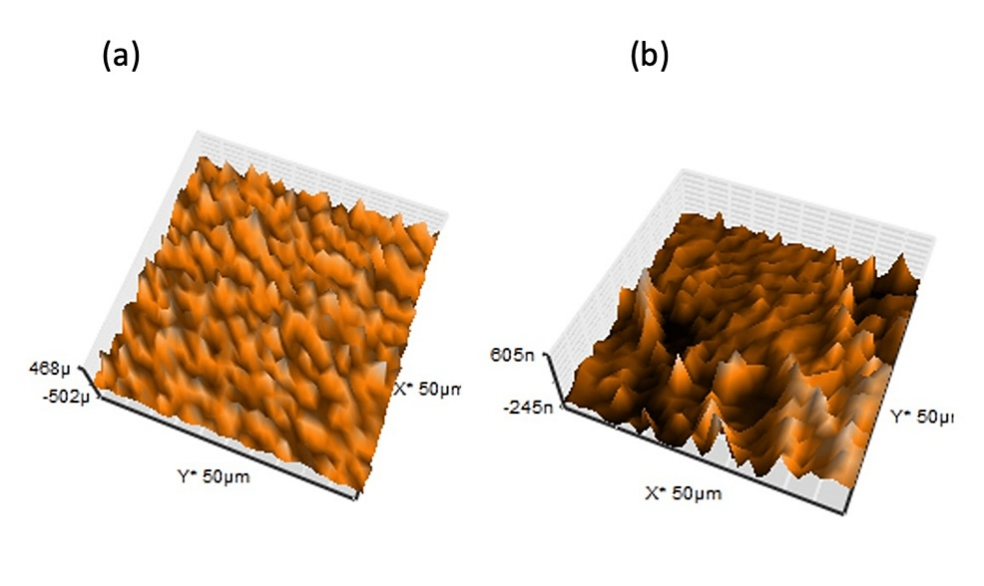
AFM image of shells showing typical surface roughness with intact periostracum shells (a) 1 and (b) 3

The AFM analysis revealed that the topography of shell 1 demonstrated a rough surface with irregularities when viewed at a greater depth, and shell 3 showed a greater roughness in topography in comparison. The mean surface roughness of periostracum intact shells was found to be 29.021 nm (Figure 4a) and 158.91 nm (Figure 4b), respectively, for shells 1 and 3. Moreover, the shells with the largest surface roughness showed wavy and ripple microstructures attributing to the antibiofilm effect.

FTIR analysis

**Figure 5:**
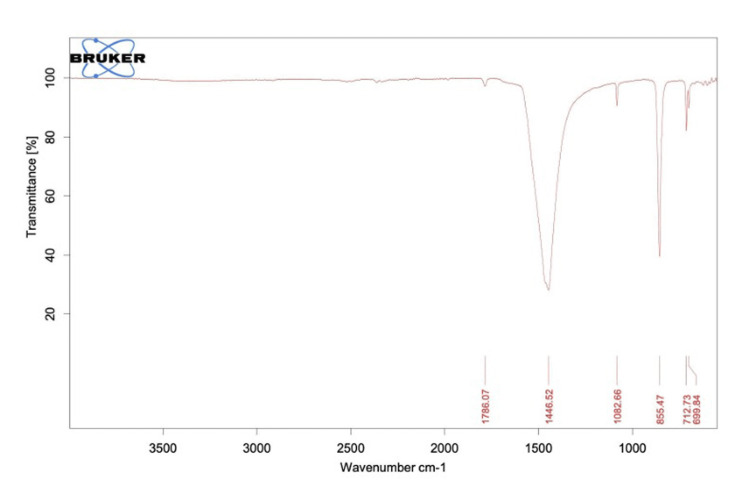
FTIR spectrum of periostracum removed from a shell

The FTIR analysis revealed the presence of sharp peaks indicative of several chemical constituents (Figure 5). These constituents include aromatic hydrocarbons, alkanes, long-chain aliphatic CH groups, ketone or aldehyde groups, lipids, and proteins. It was seen that peak 855 corresponds to alpha-glucan bonds, while 1082 corresponds to beta-glucan bonds, 1446 corresponds to the proteins and lipids group, and 1786 corresponds to the phospholipid group. In a study by Xu et al., the FTIR analysis was performed on periostracum, which revealed that the protein peaks were very strong, indicating that proteins are the primary components of fresh periostracum [14].

Surface tension measurement using a goniometer

Wetting, or the interaction of water with various surfaces, is a crucial aspect in numerous biological and technological contexts. The contact angle, formed at the interface where water, air, and the surface converge, is a fundamental parameter in understanding these interactions. As shown in Table 1, it was clear that shells with the organic membrane (OM), viz., 2 and 4, showed a contact angle of <90°, indicating a hydrophilic surface, and shells 1, 3, 5, and 6 with a contact angle of >90° attributed to a hydrophobic surface which could be due to the microstructures present on the organic matter of the shells. The measurements were taken in the presence of the organic matter, as any acid treatment could form pits, which might lead to swelling due to liquid absorption.

**Table 1:** Sessile drop method of measuring the contact angle of periostracum intact shells using a goniometer

Shells	Contact angle in degrees
S1	95.3
S2	80.14
S3	93.07
S4	24.9
S5	97.83
S6	108.53

Surface roughness

The surface roughness is a key factor that determines the surface texture. AFM gives the arithmetic mean deviation (Ra), root mean square (Rq), and maximum height (Rz) of the surface roughness profile. The roughness profile of periostracum intact shells 1 and 3 showed a Ra of 0.676 and 0.9 with their corresponding Rq values at 0.856 and 1.056, respectively (Table 2). The lower the Ra and Rq, the higher the biofilm formation, as evidenced in shell 1 (Figure 1). On the other hand, a slightly higher value of Ra and Rq in shell 3 is attributed to increased roughness, making the surface incompatible with the planktonic forms to establish adhesion and colony formation for biofilm development, as shown in Figure 1. The degree of difference in the microstructures accounting for roughness shells 1 and 3 determined the colonization efficiency of the biofilm-forming bacterium.

**Table 2:** Stylus profilometry of periostracum intact shells and their corresponding surface roughness profile, namely, mean roughness (Ra), root mean square (Rq), and the mean difference between the highest peaks and lowest valleys (Rz)

Shells	Ra	Rq	Rz
S1	0.676	0.856	4.273
S2	2.334	3.453	18.002
S3	0.9	1.056	4.175
S4	3.193	4.588	24.127
S5	1.569	2.131	13.86
S6	0.937	1.165	5.602

Statistical analysis

A two-way ANOVA was conducted to determine if there is a statistical difference between the extent of biofilm formed on the six different surfaces and the extent of biofilm formed on surfaces with intact periostracum and those with periostracum removed. The ANOVA results are shown in Table 3.

**Table 3:** ANOVA df: degrees of freedom, SS: sum of squares of errors, MS: mean sum of squares of errors, F: ratio of variants with respect to error, p: significant value (p<0.05)

Source of variation	SS	df	MS	F	p-value
Shell samples	0.0743	5	0.0148	28.1550	2.66E-09
Test and control	0.0164	1	0.0164	31.1855	9.55E-06
Interaction	0.0012	5	0.0002	0.4854	0.78366
Error	0.0126	24	0.0005		
Total	0.1047	35			

## Discussion

Shells possess a durable yet flexible proteinaceous shell covering produced by their mantle, known as the "periostracum." Scardino et al. indicated that fouling organisms prefer compromised areas of periostracum during initial attachment, and mussels with intact periostracum tend to exhibit greater resistance to fouling [7]. Various studies documented the impact of microtopographical features resembling mussel shells as a physical fouling deterrent [8]. Biomimicry, which involves replicating natural surface microtopographies, offers a means to elucidate the influence of physical surfaces without any chemical or mechanical hindrances typically associated with surfaces. Replicated surface microtopographies inspired by marine organisms also exert deterrent effects on the settlement of fouling organisms.

This study assessed the biofilm-forming or biofilm-inhibiting potential of shell architecture with and without periostracum by probing the surface roughness, contact angle, and surface texture using SEM and AFM with the presence and absence of periostracum. Seashells were initially collected from a natural urban beach in Chennai, Tamil Nadu, India, based on their colors, shapes, and unique patterns. Six pairs of seashells with various surface textures were chosen. We observed that periostracum intact shells showed lesser biofilm formation when compared with those shells without periostracum. Shells without periostracum promoted 15% more biofilm than those with intact periostracum (p<0.001).

Some studies reported on the presence of ripple-like microtopography, surface chemistry, cumulative filtration, and the production of antibiofilm chemical compounds as a defense strategy [7,15]. Scardio et al. showed that the ripple structures on *Mytilus* sp. surface significantly reduced the barnacle larvae deposition [16].

The microtopography of these shells analyzed using SEM revealed that these two extreme surface textures have a greater influence in enhancing the surface area, facilitating the adhesion of bacteria, leading to biofilm formation [17]. This often demonstrates a significant correlation between preserving the intact periostracum and decreasing adherence, augmenting the classical antibiofilm effect as evidenced in marine ecosystems [11]. It was also inferred that the shells with smooth surfaces had higher biofilm propensity than shells with impregnated micro-textured surfaces. These observations corroborated well with earlier studies highlighting the natural nanostructured surfaces and their potential involvement in killing bacteria. This contact-killing mechanism could also be one of the reasons for effectively killing the biofilm-forming bacterium, thereby inhibiting colonization [18].

Further, the AFM analysis tends to determine the surface microtopography's mean surface roughness, height, and magnitude. A significant difference exists in the mean surface roughness of periostracum intact shells (1 and 3), as shown in Figure 3. When viewed through AFM, these nanostructured surfaces are providentially attributed to the nano pillared shell surface, facilitating the streptococcal adhesion forces in the process of adhesion, detachment, and transmission across the shell surface. Further, the nano pillared surfaces determined the number of bacteria to be adhered using the force exerted by the donor [19]. The varied nanostructures/pillars on the shell surface are intended to confer mechanical properties, including elastic moduli and turgor pressure, to the bacteria to influence the biofilm formation or inhibition [20].

We also observed that the contact angle influences the bacterium's attachment and biofilm formation. The wetting properties might help the bacteria to thrive in adverse conditions correlating with distinct surface topologies, as evident from the AFM studies [21]. We observed that more hydrophobic surfaces (with intact periostracum) lead to less attachment, a negative correlation (correlation coefficient=-0.67). In addition, higher root mean square roughness (Rq) or higher maximum height (Rz) of the pillared surface lead to higher biofilm attachment (correlation coefficient = 0.536). More detailed studies are required to arrive at a definite conclusion.

The two-way ANOVA denoted a statistical difference between biofilm formed on various shell surfaces (p<0001). This is possibly due to the surface roughness and hydrophobicity of the surface. Furthermore, surfaces with and without periostracum allow various amounts of biofilms to form (p<0001), which may be due to the biochemical properties of this biopolymer present on them, which may require more detailed studies. This work aimed to determine if biomimetic surfaces could inhibit biofilm formation. By using an oral pathogen, namely *Streptococcus mutans*, this study was directed toward the possibility of preventing biofilm in dental structures.

Given the intricate nature of natural product research and the time limitations of this research program, a conclusive identification of the mechanism of action of this product as an antibiofilm agent in dentistry remains elusive. Consequently, there is a compelling need for extensive, long-term research into periostracum's chemical antifouling defense systems. This research should encompass a broader spectrum of commonly encountered oral microorganisms and include expanded field trials. Additionally, further elucidation of the chemical structure using various analytical techniques and comparisons against databanks can unveil the chemical identity of the compound responsible for the observed antifouling activity.

As this study represents a preliminary investigation, our focus has been primarily on confirming periostracum's antibiofilm properties and examining the shell's surface texture. There are several potential areas for future research: (1) Future studies can delve deeper into the development of dental varnishes and irrigants by incorporating periostracum to enhance their antibiofilm properties and potential applications in oral care. (2) A more comprehensive and detailed analysis of the shell's surface texture can be pursued. This information could be leveraged to design dental components using the principles of biomimetic dentistry, potentially leading to innovative and more effective dental materials and devices.

## Conclusions

In summary, our study demonstrated that shell surfaces coated with periostracum exhibited superior antibiofilm properties against *Streptococcus mutans* compared to periostracum-free areas. This finding underscores the potential of periostracum in preventing bacterial adhesion, suggesting the possibility of applying this concept in dentistry to develop natural products with antibiofilm properties. This study also revealed that surface topographies play a pivotal role not only in biofilm formation but also in conferring anti-adherent properties based on surface wettability. Further research is warranted to gain a deeper understanding of the role of periostracum in affecting biofilm formation, as well as its underlying composition.

## References

[1] Flemming HC, Wingender J, Szewzyk U, Steinberg P, Rice SA, Kjelleberg S (2016). Biofilms: an emergent form of bacterial life. Nat Rev Microbiol.

[2] Diaz PI (2012). Microbial diversity and interactions in subgingival biofilm communities. Front Oral Biol.

[3] Siqueira JF Jr, Rôças IN (2011). Optimising single-visit disinfection with supplementary approaches: a quest for predictability. Aust Endod J.

[4] Venkateshbabu N, Anand S, Abarajithan M, Sheriff SO, Jacob PS, Sonia N (2016). Natural therapeutic options in endodontics - a review. Open Dent J.

[5] Abarzua S, Jakubowski S (1995). Biotechnological investigation for the prevention of biofouling. I. Biological and biochemical principles for the prevention of biofouling. Mar Ecol Prog Ser.

[6] Wahl M, Kröger K, Lenz M (1998). Non‐toxic protection against epibiosis. Biofouling.

[7] Scardino A, De Nys R, Ison O, O'Connor W, Steinberg P (2003). Microtopography and antifouling properties of the shell surface of the bivalve molluscs Mytilus galloprovincialis and Pinctada imbricata. Biofouling.

[8] Bers AV, Wahl M (2004). The influence of natural surface microtopographies on fouling. Biofouling.

[9] Scardino AJ, de Nys R (2004). Fouling deterrence on the bivalve shell Mytilus galloprovincialis: a physical phenomenon?. Biofouling.

[10] Grandison C, Scardino A, Ovenden S (2011). An investigation of the antifouling potential of extracts of the periostracum of Mytilus sp.. Defence Science and Technology Organisation.

[11] Kang JY, Bangoura I, Cho JY, Joo J, Choi YS, Hwang DS, Hong YK (2016). Antifouling effects of the periostracum on algal spore settlement in the mussel Mytilus edulis. Fish Aquatic Sci.

[12] Farag EAA, Morsi TS, Wahsh MM, El-Etreby AS (2021). Resin nano-ceramic material surface roughness, color stability, and bacterial adhesion evaluation after different scaling methods. Braz Dent Sci.

[13] Barac R, Gasic J, Trutic N (2015). Erosive effect of different soft drinks on enamel surface in vitro: application of Stylus profilometry. Med Princ Pract.

[14] Xu J, Zhang G (2014). Biogenic nanospheres of amorphous carbonated Ca-Mg phosphate within the periostracum of the green mussel Perna viridis. J Struct Biol.

[15] Bers AV, D'Souza F, Klijnstra JW, Willemsen PR, Wahl M (2006). Chemical defence in mussels: antifouling effect of crude extracts of the periostracum of the blue mussel Mytilus edulis. Biofouling.

[16] Scardino AJ, de Nys R (2011). Mini review: biomimetic models and bioinspired surfaces for fouling control. Biofouling.

[17] Romanin M, Crippa G, Ye F (2018). A sampling strategy for recent and fossil brachiopods: selecting the optimal shell segment for geochemical analyses. Riv Ital Ig.

[18] Ivanova EP, Hasan J, Webb HK (2012). Natural bactericidal surfaces: mechanical rupture of Pseudomonas aeruginosa cells by cicada wings. Small.

[19] Hizal F, Choi CH, Busscher HJ, van der Mei HC (2016). Staphylococcal adhesion, detachment and transmission on nanopillared Si surfaces. ACS Appl Mater Interfaces.

[20] James SA, Powell LC, Wright CJ (2016). Atomic force microscopy of biofilms—imaging, interactions, and mechanics. Microbial biofilms.

[21] Werb M, Falcón García C, Bach NC, Grumbein S, Sieber SA, Opitz M, Lieleg O (2017). Surface topology affects wetting behavior of Bacillus subtilis biofilms. NPJ Biofilms Microbiomes.

